# Mercury Exposure and Risk of Type 2 Diabetes: A Systematic Review and Meta-Analysis

**DOI:** 10.1155/2022/7640227

**Published:** 2022-09-02

**Authors:** Behnam Ghorbani Nejad, Tahereh Raeisi, Parisa Janmohammadi, Fatemeh Mehravar, Mahtab Zarei, Azadeh Dehghani, Niki Bahrampour, Mohammad Hosein Darijani, Fatemeh Ahmadipour, Mohammad Mohajeri, Shahab Alizadeh

**Affiliations:** ^1^Department of Toxicology and Pharmacology, Faculty of Pharmacy, Kerman Medical University, Kerman, Iran; ^2^Department of Medicine, Hormozgan University of Medical Sciences, Bandar Abbas, Iran; ^3^Department of Clinical Nutrition, School of Nutritional Sciences and Dietetics, Tehran University of Medical Sciences, Tehran, Iran; ^4^Department of Epidemiology and Biostatistics, School of Public Health, Tehran University of Medical Sciences (TUMS), Tehran, Iran; ^5^Department of Cellular and Molecular Nutrition, School of Nutritional Sciences and Dietetics, Tehran University of Medical Sciences, Tehran, Iran; ^6^Nutrition Research Center, Department of Community Nutrition, Faculty of Nutrition and Food Science, Tabriz University of Medical Sciences, Tabriz, Iran; ^7^Department of Nutrition, Science and Research Branch, Islamic Azad University (SRBIAU), Tehran, Iran

## Abstract

**Methods:**

Scopus and PubMed databases were systematically searched from their inception to November 2021 to obtain pertinent studies. Standardized mean differences (SMDs) with corresponding 95% confidence intervals (CIs) were calculated to evaluate the difference in Hg levels between people with and without T2DM. The association of the Hg exposure with T2DM was assessed using a random-effects model by pooling the odds ratios (ORs) and 95% CIs.

**Results:**

A total of 17 studies, with 42,917 participants, aged ≥18 years, were analyzed. Overall, Hg levels were significantly higher in T2DM patients compared with non-T2DM controls (SMD = 1.07; 95%CI = 0.59 to 1.55, *P* ≤ 0.001), with significant heterogeneity across studies (I^2^ = 96.1%; *P*=≤0.001). No significant association was found between Hg exposure and risk of T2DM in the overall analysis and subgroup analysis based on the source of sample and study design. However, higher exposure to Hg was related to reduced risk of T2DM in men (OR = 0.71; 95%CI = 0.57 to 0.88), but not in women. No significant evidence for publication bias was detected.

**Conclusions:**

Although the Hg level in T2DM is significantly higher than that of nondiabetics, there was no association between Hg exposure and the overall risk of T2DM. Nevertheless, our study shows that higher exposure to Hg might reduce the risk of T2DM in men.

## 1. Introduction

Type 2 diabetes mellitus (T2DM) is a worldwide health concern [1]. This disease has put considerable economic burden on health systems globally that is forecasted to be increased even more in the future [2]. It is a multifactorial disease with different etiologies ranging from genetics to lifestyle [3] and is linked to further complications such as cardiovascular and renal diseases, as well as mortality [4]. The effect that exposure to heavy metals can have on T2DM [5, 6], obesity [7, 8], and metabolic syndrome [9] has been assessed previously. Specifically, some metals such as cadmium (Cd), Hg, and metalloid arsenic (As) are hypothesized to be related to the incidence of T2DM [5]. However, the available evidence is contradictory. The sources of these toxic metals are mainly contaminated water, polluted air, crops harvested in contaminated soil, dental care, fish consumption, and some industrial processes [10].

Hg is a heavy metal known for toxicity that exists in several forms. Inorganic Hg includes elemental or metallic mercury (Hg^0^) and mercurous (Hg_2_^++^) or mercuric (Hg^++^) salts, while organic Hg includes compounds in which Hg is bonded to a structure containing carbon atoms (ethyl, methyl, phenyl, etc.) [11]. The biological behavior and toxicity of these forms vary considerably [11]. In general, Hg exposure has a broad range of toxic effects on cardiovascular, pulmonary, hematological, digestive, renal, immune, nervous, endocrine, and reproductive systems [12]. In relation to diabetes, this toxic agent can target *β*-cells in the pancreas and induces dysfunction and apoptosis [13]. Several mechanisms are introduced such as altering Ca^2+^ homeostasis, activation of phosphatidylinositol 3-kinase (PI3K) Akt signaling pathway, and reactive oxygen species (ROS) production [5]. Some studies have assessed the presence of this metal in diabetic patient's scalp hair [14], urine [15], and blood [16–19] to examine any relationships between its levels with T2DM markers, but the results were heterogeneous. While some studies demonstrated positive relationships between T2DM and Hg levels in blood [20–25], urine [26], hair [27], and toenail [28], some other studies did not observe any relationship [29–36]. These discrepancies might be due to the differences in the population characteristics and sources of exposure (urine, blood, and nail). We, therefore, aimed to summarize the relationship between Hg levels in different body samples with the risk of T2DM in a comprehensive systematic review and meta-analysis.

## 2. Methods

In order to design and implement the present study, the guidelines of the Statement of Systematic Reviews and Preferred Reporting Meta-Analysis (PRISMA) have been considered and followed [37].

### 2.1. Search Strategy

The online databases PubMed and Scopus were searched extensively for related articles published before November 2021. A different combination of keywords was used in the search, which is listed as follows: ((((((“Mercury”[Mesh]) OR (Mercury[Title/Abstract])) OR (methyl Mercury[Title/Abstract])) OR (Quicksilver[Title/Abstract])) OR (dimethylmercury[Title/Abstract])) OR (“colloidal Mercury”[Title/Abstract])) AND (((((“Diabetes Mellitus, Type 2”[Mesh]) OR (diabetes[Title/Abstract])) OR (“type 2 diabetes mellitus”[Title/Abstract])) OR (T2DM[Title/Abstract])) OR (“noninsulin-dependent diabetes mellitus”[Title/Abstract])). Only English studies were reviewed. All review articles and study references were checked to minimize the possibility of losing studies. To speed up the screening process, all identified studies were imported into an EndNote library, and duplicates were removed. The selection of eligible studies was independently reviewed by two researchers. First, the titles and abstracts of the studies were evaluated, and then the full text of the remaining publications was independently reviewed.

### 2.2. Eligibility Criteria

The criteria for including eligible articles in this systematic review and meta-analysis were defined as follows: (A) cohort studies, cross-sectional, or case-control studies, (B) having a nondiabetic control group, (C) studies that reported odds ratios (ORs), hazard ratios (HRs), or relative risks (RRs) and the corresponding 95% confidence interval (CI) for the association of the Hg exposure in the blood, urine, hair, and nails with T2DM, (D) studies reported the mean and standard deviation (SD) of Hg in patients with T2DM and healthy controls, (E) the full text of the article was available in English, and (F) studies were conducted on adults (aged ≥18 years). Case reports, book chapters, conference papers, letters, editorial papers, and animal and cell culture studies were excluded.

### 2.3. Data Extraction

Two researchers independently reviewed the full text of the studies and extracted the data and resolved the differences through discussion with the third independent researcher. The following information was extracted: (1) study characteristics (name of the first author, design of the study, year of publication, country/geographical location, target population, number of participants, duration of study, gender), (2) mean and SD of Hg, and (3) relevant reported risk estimates (including ORs, RRs, HRs) and the corresponding 95% CIs.

### 2.4. Quality Assessment of Studies

In this meta-analysis, the Newcastle–Ottawa Scale (NOS) questionnaire was used to evaluate the quality of studies in three areas (study selection, study group comparison, and exposure assessment) [38]. Studies with scores of 4 or more were considered a medium to high-quality studies.

### 2.5. Statistical Analysis

In this meta-analysis, a random effects model was used to test the effect of interest. Mean differences in Hg levels between the patients with T2DM and healthy controls were reported as standardized mean difference (SMD) and 95%CI. Standardized mean differences (SMD) for each original study were derived using the method of Cohen's d [39] as the difference between means divided by the pooled standard deviation. Risk estimates reported for the relationship of Hg exposure to T2DM were pooled to estimate the overall effect size (OR and 95%CI). Heterogeneity across studies was assessed with *I*^2^ statistics. Heterogeneity was considered significant if *I*^2^ >50% (*P* < 0.1). Funnel diagrams and Egger's test were used to assess publication bias. Subgroup analysis was performed according to the type of study (prospective cohort vs. case-control), gender (both males and females), and sample source (blood, urine, and nails). STATA (version 14.0; Stata Corporation, College Station, TX) was used to perform all statistical tests for the current meta-analysis. *P* value <0.05 was considered statistically significant for all statistical analyzes.

## 3. Results

### 3.1. Study Characteristics

**Figure 1:**
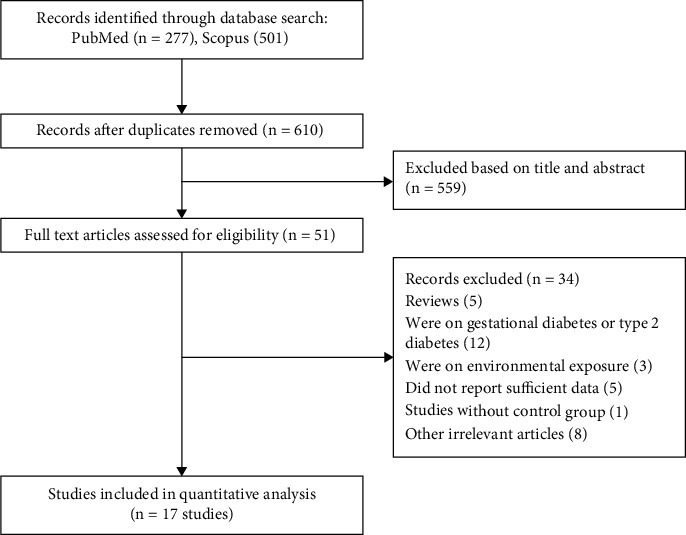
Flowchart of the study.

The systematic search of databases yielded a total of 778 studies. After excluding duplicate publications (*n* = 168) and unrelated studies by titles/abstracts (*n* = 559), 51 publications underwent full-text screening, of which 17 studies [14–16, 18, 19, 28, 29, 32–36, 40–44], with a total sample size of 42,917 participants (5,545 cases of T2DM), published between 1984 and 2021, were eligible to be included in the current meta-analysis according to the inclusion criteria. The flowchart reporting the process of screening is presented in Figure 1. Some included studies reported different effect sizes based on their studied subgroups; for these studies, we extracted and analyzed all suitable effect sizes. Of the included studies, data on Hg levels in T2DM patients vs. healthy controls were reported in 10 studies with 15 effect sizes [14–16, 19, 32, 33, 40–42, 44], and 11 studies (3 prospective cohorts and 8 case-control) with 17 effect sizes [15, 18, 28, 29, 32, 34–36, 42–44] reported risk estimates for the association between Hg exposure and the risk of T2DM. All studies reporting risk estimates for T2DM adjusted the analysis for the potential confounders. The sample size of the analyzed studies ranged between 53 and 15,327 subjects. Regarding the sex of participants, 5 studies reported data for subgroups of males and females separately [14, 18, 35, 36, 41], 1 study just included males [19], 1 study just included females [15], and the remaining studies were performed on a combination of both genders. The quality of studies was medium to high, with scores ranging from 4 to 9. The characteristics of the analyzed publications are presented in Table 1.

### 3.2. Mercury Levels in T2DM

**Figure 2:**
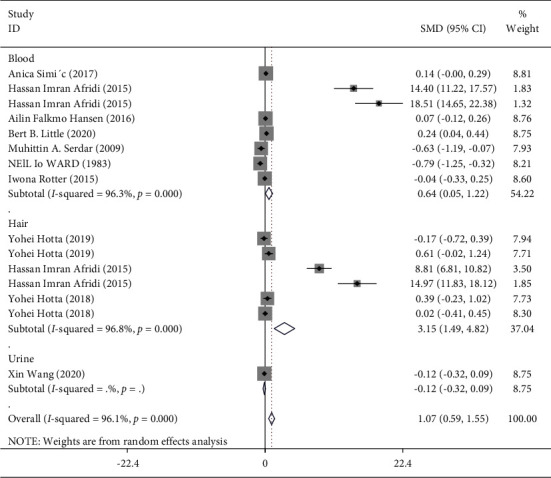
Forest plot for the mean levels of Hg in patients with T2DM compared with healthy controls stratified by the type of sample.

In the pooled analysis of all eligible studies, Hg levels were significantly higher in T2DM patients compared with non-T2DM controls (random effects, SMD = 1.07; 95% CI = 0.59 to 1.55, *P* ≤ 0.001) (Figure 2), with a significant heterogeneity across studies (I2 = 96.1%; *P*=≤0.001). In the stratified analysis by type of sample, compared with healthy controls, Hg levels were also significantly higher in blood (7 studies [16, 19, 32, 40–42, 44], SMD = 0.64 *μ*g/L; 95% CI = 0.05 to 1.22, *P*=0.03) and hair (3 studies [14, 33, 41], SMD = 3.15 *μ*g/L; 95% CI = 1.49 to 4.82, *P* ≤ 0.001) of patients with T2DM, but not in urine samples (based on 1 study [15]) (Figure 2).

### 3.3. Mercury and Risk of T2DM

**Figure 3:**
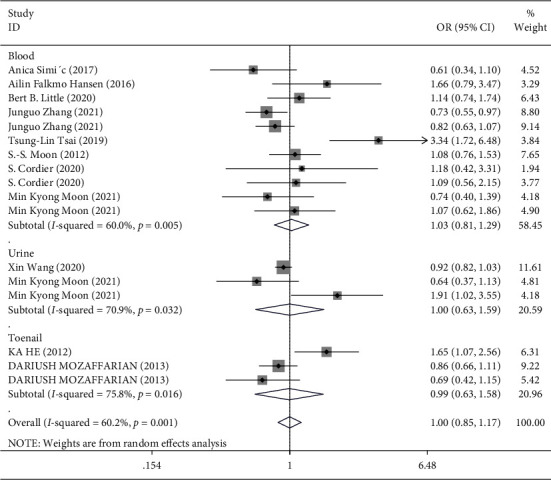
Forest plot for the association between Hg and risk of T2DM stratified by the type of sample.

**Figure 4:**
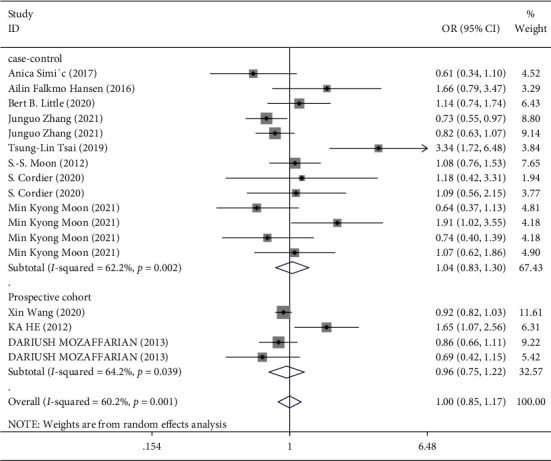
Forest plot for the association between Hg and risk of T2DM stratified by the study design.

**Figure 5:**
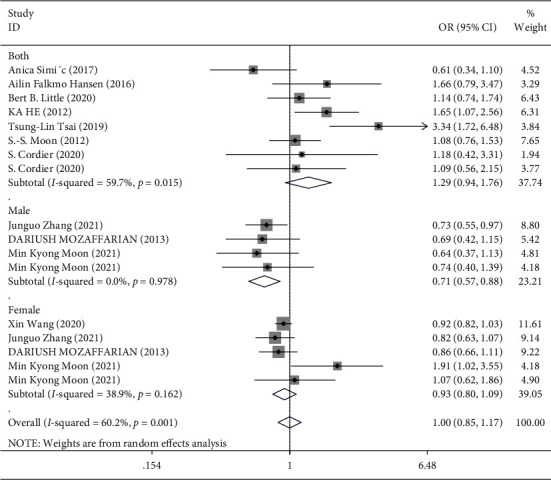
Forest plot for the association between Hg and risk of T2DM stratified by the sex of participants.

**Figure 6:**
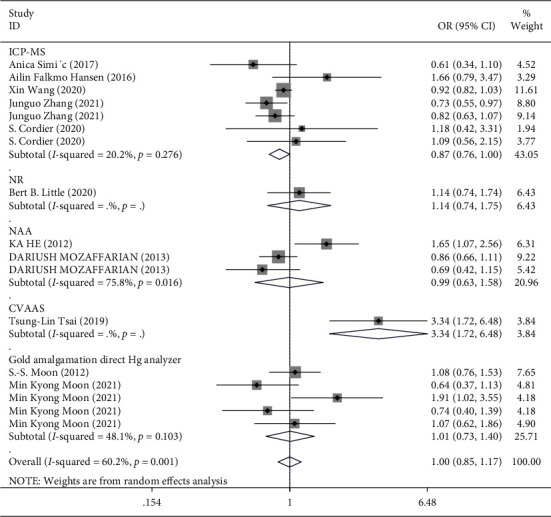
Forest plot for the association between Hg and risk of T2DM stratified by the method used for the measurement of Hg. ICP-MS: inductively coupled plasma mass spectrometry, NR: not reported, NAA: neutron-activation analysis, CVAAS; cold vapor atomic absorption spectrometry.

In the overall analysis and subgroup analysis based on the source of the sample (blood, urine, and toenail) (Figure 3) and study design (case-control vs. prospective cohort) (Figure 4), no significant association was found between Hg exposure and risk of T2DM. The pooled effect size for case-control studies was (SMD = 1.04; 95% CI = 0.83 to 1.30), and for cohort studies, it was (SMD = 0.96; 95% CI = 0.75 to 1.22), indicating no association between Hg and T2DM in both case-control and cohort studies (Figure 4). However, in the stratified analysis by the sex of participants, it was observed that higher exposure to Hg might be related to a reduced risk of T2DM in males (3 studies [18, 35, 36], OR = 0.71; 95% CI = 0.57 to 0.88), but not in females (Figure 5). Moreover, in the stratified analysis by the method used for the measurement of Hg, overall Hg exposure was not associated with T2DM (Figure 6).

### 3.4. Publication Bias

**Figure 7:**
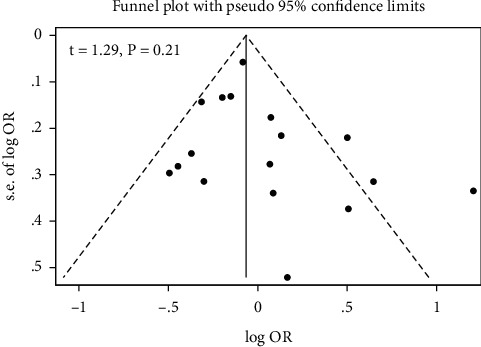
Funnel plot for publication bias in studies exploring the relation of Hg to the risk of T2DM.

Egger test and funnel plot detected no significant evidence for publication bias in studies investigating the relation of Hg exposure to the risk of T2DM (Figure 7).

## 4. Discussion

The current study is the first systematic review and meta-analysis which aimed to investigate the association of Hg levels in different body samples with the risk of T2DM. Overall, we found consistent epidemiological evidence that Hg levels in the blood and hair samples of diabetic patients were considerably higher than in the nondiabetic control group. Nevertheless, overall, the findings of this study revealed no significant association between Hg exposure and the risk of T2DM. However, Hg exposure in the male subgroup might reduce the risk of T2DM.

The findings of the current meta-analysis demonstrated no significant association between Hg exposure and the risk of T2DM. This lack of association was even observed in the subgroups by sample source (blood, urine, and toenail) and the type of study (case-control vs. prospective cohort). A cross-sectional study conducted on 1588 men and 1596 women with age ≥30 years in the general population in South Korea indicated that blood Pb, Hg, and Cd concentrations in diabetic patients were slightly higher than the nondiabetic individuals; this difference, nonetheless, was not significant [34]. Even after controlling for age, gender, location, smoking, alcohol use, and regular exercise, the prevalence of diabetes was not affected by the blood heavy metals concentrations [34]. However, in the study by Tsai et al., in 2019 on 646 Taiwanese adults, Hg levels in red blood cells (RBC-Hg) of T2DM patients were considerably higher than the nondiabetic subjects. After controlling for the potential confounders, a significant direct association was reported between the RBC-Hg and the prevalence of T2DM [43]. A cohort study on toenail samples of 9262 American subjects showed that toenails' Hg concentration did not lead to a higher incidence of diabetes in women and men [18]. This finding remained constant even after separate analyzes based on classifications of Hg with higher concentrations, fish or Omega 3 consumption, BMI, and age. A review study, including 34 in vivo and in vitro studies, showed a probable association between the total Hg concentration and the risk of diabetes. However, sufficient evidence for a causal and consistent relationship did not exist [45]. A case-control study showed a considerable increase in fasting blood glucose levels among individuals with high blood Hg levels (>16 mg/L) [29]. Moreover, supporting our findings, considering Hill's causal criteria, a systematic review of 29 publications did not show sufficient evidence of any associations between Hg levels and diabetes [46]. The discrepancy among studies may result from study design, source of Hg exposure, or population characteristics such as age and sex. Moreover, because blood levels of Hg differ among ethnic groups [34], the heterogeneity in the results might be due to differences in ethnicity. The levels of Hg exposure are also dependent on the geographic region [47], and a source of the inconsistency of the available evidence may be differences in the geographic region of various studies.

Although, in the overall analysis, the current study demonstrated no association between Hg exposure and the risk of diabetes, the results of the gender-based analysis revealed that more exposure to Hg among the subgroup of men might be associated with a lower risk of T2DM. This finding resulted from the pooled analysis of only three studies [18, 35, 36]; thus, this conclusion should be interpreted with caution; however, our findings were comparable to a large case-control study (15327 subjects) by Zhang et al. which revealed a reverse association between the total Hg concentration and blood methylmercury and diabetes among adults [18]. Also, in Health Professionals Follow-Up and Nurses' Health Study, toenails' higher Hg levels were significantly associated with lower incidence of diabetes in both genders [35]. The mechanisms underlying the likely inverse relationship between Hg exposure and lower incidence of diabetes in men are still unknown. Previous studies reported the significance of oxidative stress in the pathogenesis of Hg toxicity [48]. As a compensatory mechanism against oxidative stress, Hg could increase the gene expression of proteins with antioxidant activity, including catalase, copper, zinc-superoxide dismutase, glutamate-cysteine ligase, thioredoxin reductase 1, manganese-superoxide dismutase, and can stimulate the antioxidant signaling pathway via direct interaction with the cysteine residues of the Keap1 and/or Akt/glycogen synthase kinase 3 beta/Fyn pathway [48], which the mentioned mechanism is protective against T2DM [49]. Also, some studies have found a reverse association between Hg exposure and cardiovascular disease [50–52] which might be related to the aforementioned cellular reparative function against Hg-induced oxidative stress. Although, this inverse association might be due to confoundings, such as relationships between higher Hg exposure and other factors that reduce the risk of T2DM. The included studies adjusted for potential covariates, but residual confounding because of unmeasured factors, particularly for fish consumption which is a main source of Hg exposure [18] cannot be excluded. Despite this, the results of the majority of the included studies were adjusted for fish consumption. Omega-3 fatty acids might attenuate Hg-induced adverse effects by improving the acute phase response and antioxidant status [53]. The observed reduced risk of T2DM with higher Hg exposure in men may also be due to higher intakes of omega-3 fatty acids [18]; thus, this finding needs confirmation and should be interpreted with caution.

The current study is the first meta-analysis examining the association between blood, hair, toenail, and urine Hg levels and the risk of T2DM. As strength, no evidence for publication bias was identified. However, some limitations of this meta-analysis should be declared. First, significant heterogeneity was found across studies; we identified that this heterogeneity did not originate from the sample type or study design, but the gender of participants. Second, the majority of the included studies were case-control in design that might be affected by the unexamined confounding factors and suffer from a higher probability of bias than cohort studies. Other weaknesses of the present study include the difference in Hg concentration of various body samples, the difference in the methods of measuring Hg levels, and the biomarkers' measurement precision. Hg levels in hair, nail, and urine are usually reflective of longer aggregation, and its levels in the blood sample are usually indicative of short-term exposure, which this could be a source of the observed heterogeneity [54].

Overall, this meta-analysis indicated that although the Hg level in diabetic individuals is significantly higher than the nondiabetics, there existed no association between Hg exposure and the risk of T2DM. However, exposure to Hg in men might reduce the risk of T2DM; however, additional studies are required to confirm this finding.

## Figures and Tables

**Table 1:** The characteristics of the included studies in meta-analysis.

Study	Country	Year	Study design	Sex	Total sample size	N cases with T2DM (age)	Sample source	Mean ± SD Hg in controls	Mean ± SD Hg in cases (T2DM)	Hg assessment	Type of effect size	
Anica Simi´c	Norway	2017	Case-control	Both	874	267 (65.4 ± 10.6)	Blood	3.19 ± 1.35	3.6 ± 4.8	ICP-MS	Mean of Hg in T2DM vs. controls OR for T2DM	Adjusted for BMI, waist-to-hip ratio, first-degree family history of diabetes, smoking habits, area, education and economic status, fat fish intake

Yohei Hotta	Japan	2019	Case-control	Male	50	27 (66.1 ± 13.2)	Hair	2.5 ± 2.94	2.12 ± 1.49	ICP-MS	Mean of Hg in T2DM vs. controls	—
				Female	46	15 (68.0 ± 8.5)		1.37 ± 1.03	2.19 ± 1.83			

Hassan Imran Afridi	Pakistan	2015	Case-control	Male	43	25 (ranged 30–50 years)	Hair	1.02 ± 0.07	1.69 ± 0.08	ICP-atomic emission spectrophotometer	Mean of Hg in T2DM vs. controls	—
							Blood	0.85 ± 0.08	1.92 ± 0.07			
				Female	47	23 (ranged 30–50 years)	Hair	0.98 ± 0.03	1.78 ± 0.07			
							Blood	0.85 ± 0.04	1.79 ± 0.06			

Ailin Falkmo Hansen	Norway	2016	Case-control	Both	883	755 (65.2 ± 10.3)	Blood	3.18 ± 5.26	3.47 ± 4	ICP-MS	Mean of Hg in T2DM vs. controls OR for T2DM	Age, sex, body mass index, waist-to-hip ratio, education, income, smoking, and family history of diabetes

Yohei Hotta	Japan	2018	Case-control	Both	Group 1:71	Group 1:12 (ranged 36–86 years)	Hair	1.94 ± 2.11	2.88 ± 3.52	ICP-MS	Mean of Hg in T2DM vs. controls	—
					Group 2:92	Group 2:33 (ranged 36–86 years)		1.94 ± 2.11	1.98 ± 1.55			

Bert B. Little	USA	2020	Case-control	Both	875	109 (55.0 ± 11.4)	Blood	0.05 ± 0.27	0.12 ± 0.41	NR	Mean of Hg in T2DM vs. controls OR for T2DM	Age, gender, smoking tobacco, duration of residence, smelter worker, blood lead level, blood arsenic, cadmium level, gamma-glutamyl transpeptidase, hypertension
Muhittin A. Serdar	Turkey	2009	Case-control	Both	53	31 (59 ± 9)	Blood	1.53 ± 0.69	1.15 ± 0.54	ICP-MS	Mean of Hg in T2DM vs. controls	—

Xin Wang	USA	2020	Prospective cohort (17 years of follow-up)	Female	1237	102 (50.0 ± 3.1)	Urine	1.23 ± 1.31	1.08 ± 1.05	ICP-MS	Mean of Hg in T2DM vs. controls HR for T2DM	Age, race/ethnicity, study site, specific gravity, education, household income, body mass index, waist circumference, smoking, alcohol consumption, physical activity, energy intake, menopausal status, and use of the hormone, seafood, and rice intake

NElL Io WARD	England	1983	Case-control	Both	85	55 (59.7 ± 10.0)	Blood	15 ± 5	12 ± 3	Neutron-activation analysis (NAA) and electrothermal atomic absorption spectroscopy (EAAS) methods	Mean of Hg in T2DM vs. controls	—

Iwona Rotter	Poland	2015	Case-control	Male	313	55 (61.3 ± 6.3)	Blood	4.53 ± 2.23	4.45 ± 1.58	Inductively coupled argon plasma optical emission spectrometry (ICP OES)	Mean of Hg in T2DM vs. controls	—

Junguo Zhang	China	2021	Case-control	Both	15327	2132 (49.75 ± 17.88)	Blood	—	—	Inductively coupled plasma dynamic reaction cell mass spectrometry	OR for T2DM	Age, sex, hypertension, poverty-income ratio, education, marital status, and daily intakes of protein, total fat, sugar, fiber, total energy, alcohol, vitamin C, vitamin B6, selenium, calcium and omega-3 polyunsaturated fatty acid, moderate recreational activities, cotinine, and estimated glomerular filtration rate
KA HE	USA	2012	Prospective cohort (18 years of follow-up)	Both	4163	288 (aged 20–32 years)	Toenail	—	—	Instrumental neutron-activation analysis	HR for T2DM	#Model 6: model 5 with additional adjustment for toenail selenium (quintiles)

Tsung-Lin Tsai	Taiwan	2019	case-control	Both	646	56 (55.37 ± 12.87)	Blood	—	—	Cold vapor atomic absorption spectrometry	OR for T2DM	Age, sex, BMI, education, hypertension, total cholesterol, fasting glucose, cigarette smoking, alcohol consumption, saltwater ﬁsh consumption, total calorie intake, protein and fat intake, geographical strata, seasonality, C-reactive protein, and hemoglobin

S.-S. Moon	South Korea	2012	Case-control	Both	3184	333 (58.8 ± 10.9)	Blood	—	—	Gold-amalgam collection method with DMA-80	OR for T2DM	Adjusted for age, sex, region, smoking, alcohol consumption, and regular exercise

DARIUSH MOZAFFARIAN	USA	2013	Prospective cohort (follow-up of 7.0 years)	Female	9267	1010 (61.2 ± 8.9)	Toenail	—	—	Neutron-activation analysis	HR for T2DM	Adjusted for age, sex, race, region, month of toenail return, family history of diabetes, smoking status, BMI, hypertension, hypercholesterolemia, future cardiovascular disease case-control status (case or control), physical activity, alcohol use, and ﬁsh consumption
				Male								

S. Cordier	Canada	2020	Case-control	Both	1874	217 (32.9 ± 4.8)	Blood	—	—	ICP-MS	OR for T2DM	Age, sex, waist circumference, smoking, omega-3 PUFAs

Min Kyong Moon	South Korea	2021	Case-control	Both	3787	NR (aged ≥19 years)	Urine and blood	—	—	Amalgamation direct Hg analyzer	OR for T2DM	Age, sex, cigarette smoking, alcohol drinking, exercise, and education levels were included as covariates

NR: not reported; T2DM: type 2 diabetes; OR = odds ratio; BMI = body mass index; ICP-MS: inductively coupled plasma mass spectrometry; PUFAs: Polyunsaturated fatty acids.

## Data Availability

All data and codes are available upon request to the corresponding author.
